# Sodium perchlorate in the space group *Pnma*. Corrigendum

**DOI:** 10.1107/S2056989017006247

**Published:** 2017-05-31

**Authors:** Gui-Ling Zhang, Yan-Tuan Li, Zhi-Yong Wu, Yu-Lan Song

**Affiliations:** a Marine Drug and Food Institute, Ocean University of China, 266003 Qingdao, People’s Republic of China

## Abstract

In the paper by Zhang *et al.* [*Acta Cryst.* (2006), E**62**, i150–i151], the sodium cation was assigned incorrectly.

## Abstract 2

Erratum to *Acta Cryst.* (2006), E**62**, i150–i151.

The authors of Zhang *et al.* (2006 ▸) have indicated that the sodium cation in the reported complex was assigned incorrectly and is in fact an ammonium cation. It is suspected that the ammonium ions originated from the commercially obtained cucurbit[5]uril starting material. The structure of ammonium perchlorate has been published on previous occasions, *e.g.* Choi *et al.* (1976 ▸).
